# Can diabetes prevention programmes be translated effectively into real-world settings and still deliver improved outcomes? A synthesis of evidence

**DOI:** 10.1111/dme.12018

**Published:** 2012-12-13

**Authors:** M Johnson, R Jones, C Freeman, H B Woods, M Gillett, E Goyder, N Payne

**Affiliations:** University of Sheffield, School of Health and Related Research (ScHARR)Sheffield, UK

## Abstract

**Objective:**

Randomized trials provide evidence that intensive lifestyle interventions leading to dietary and physical activity change can delay or prevent Type 2 diabetes. Translational studies have assessed the impact of interventions based on, but less intensive than, trial protocols delivered in community settings with high-risk populations. The aim of this review was to synthesize evidence from translational studies of any design to assess the impact of interventions delivered outside large randomized trials.

**Methods:**

Medical and scientific databases were searched using specified inclusion and exclusion criteria. Studies were included that used a tested diabetes preventive study protocol with an adult population at risk from Type 2 diabetes. Included papers were quality assessed and data extracted using recommended methods.

**Results:**

From an initial 793 papers, 19 papers reporting 17 studies were included. Translational studies from a range of settings utilized a variety of methods. All were based on the US Diabetes Prevention Programme protocol or the Finnish Diabetes Prevention Study, with modifications that increased feasibility and access. The main outcome that was reported in all studies was weight change. Weight loss, which occurred in all but one study, was greater in intervention arms than in control subjects. No consistent differences were found in blood glucose or waist circumference.

**Conclusions:**

Translational studies based on the intensive diabetes prevention programmes showed that there is potential for less intensive interventions both to be feasible and to have an impact on future progression to diabetes in at-risk individuals.

## Introduction

Prevention of Type 2 diabetes is a major global public health objective, with 366 million people estimated to have the condition worldwide, and the anticipation that this will increase to 522 million by 2030 [1]. Individuals who have raised blood glucose levels, yet are below the threshold for Type 2 diabetes, are estimated to have between 5 and 15% greater absolute risk for progressing to diabetes than those with normal glucose levels [2]. As it is known that modifiable risk factors such as obesity can be prevented or reversed by changing lifestyle behaviours (in particular, dietary and physical activity), there is an opportunity to intervene to prevent or delay diabetes onset.

A number of randomized controlled trials of intensive lifestyle change have shown that changes in both dietary and physical activity behaviours can achieve positive results [3–7]. Both the Diabetes Prevention Program (DPP) based in the USA [3,8], and the smaller Diabetes Prevention Study (DPS) in Finland [4,9] achieved a reduction of diabetes incidence compared with the control groups.

The proven effectiveness of these two trials has given urgency to the question of whether such effects might be replicated in community settings, including primary care. ‘Translational research’ has been described as the assessment of smaller programmes in ‘real-world’ settings, where resources are more limited and samples less selective than in the trial environment [10].

Ali and colleagues examined and meta-analysed 28 translational studies based on the DPP, including studies where the sample included no more than 50% of people with Type 2 diabetes. The authors reported a 4% weight loss across a range of interventions at 12 months' follow-up [11].

Our review differs in that we provide a narrative synthesis of ‘translational’ studies based on both the DPP and the DPS diabetes prevention protocols. We excluded studies that had knowingly recruited individuals with a diagnosis of Type 2 diabetes.

Our aim was to assess ‘real-world’ lifestyle intervention programmes of any design to prevent Type 2 diabetes and/or reduce BMI and weight in high-risk adults. We aimed to compare reported effectiveness with that of larger trials and assess the modification of components and design.

## Methods

Methods were agreed with the National Institute for Health and Clinical Excellence (NICE), who funded the study, and were carried out in line with their methods manual [12]. Documents relating to the scope of the review are available online at http://guidance.nice.org.uk/PHG/45.

### Inclusion criteria

Studies of any design with any length of follow-up were included. The population assessed were adults at risk from Type 2 diabetes, and with raised blood glucose levels. Only lifestyle interventions based on protocols that were replicable and that had been shown to have some success in preventing or delaying Type 2 diabetes were included. Any comparator was considered for inclusion, and the primary outcomes were diabetes incidence, as well as changes in weight, BMI and waist circumference.

### Exclusion criteria

Studies were excluded if they did not state the protocol on which the intervention was based, or if they included individuals that were under the age of 18 years, or were known to have a diagnosis of Type 1 or Type 2 diabetes.

### Searching

Searches were undertaken by a qualified information specialist. The electronic databases MEDLINE, MEDLINE In-Process, EMBASE, CINAHL, British Nursing Index and Archive, The Cochrane Library, Science Citation Index, Social Science Citation Index, PsycINFO and selected EPPI Centre Databases were searched.

An initial overarching search was undertaken at the outset of the programme of reviews. This search was generated by identifying concepts from the programme scope and from studies identified from key known literature as being relevant to the review questions; free text and Medical Subject Headings (MeSH) terms were then devised.

The searches were limited to English language publications because of lack of resources for translation. Diabetes prevention translational studies are a relatively recent development (during the last two decades), following on from the larger trials. Therefore, the search was limited to articles published between 1990 and 2011(see also Supporting Information, Appendix S1).

We searched the reference lists of included papers as well as reviews that were identified in the searches. Topic experts, including members of the NICE Programme Development Group, were asked to identify relevant articles and studies.

### Assessment for inclusion

Search results were transferred to a reference management database and three reviewers (MJ, RJ, CF) each sifted one third of the titles (and, if necessary, abstracts) for relevance to the review question. The sifting of each third of the results was double-checked by a different reviewer (MJ, RJ, CF). Full texts were retrieved of papers that were assessed as relevant and these were discussed in meetings between the three reviewers to finalize the set of included papers. Disagreements were resolved by further reading of the full text to ensure relevance to the question.

### Quality assessment and data extraction

Quality of retrieved papers was assessed using a 27-item tool for the assessment of quantitative studies recommended in the NICE methods manual [12] (see also Supporting Information, Appendix S1). A single checklist was recommended for all studies with quantitative outcomes rather than a range of critical appraisal tools for each study design [12, page 202].

A data extraction form recommended for quantitative studies in the methods manual [12] was adapted for use with a range of study types. The structure of the adapted extraction form was agreed with the study funders prior to use. We extracted details of the article such as author, date and journal. Study details that were extracted included study type, population characteristics, sample size, funding, ethical considerations and intervention characteristics. For this review, we extracted data that specifically related to differences between the study and the DPP or DPS. Finally, results were extracted in terms of included outcome measures and follow-up. Incidence of Type 2 diabetes, changes in blood glucose measures, and changes in weight, BMI and waist circumference were recorded. Quality assessment and data extraction was carried out by reviewers (MJ; RJ; CF), who each double-checked a proportion of another reviewer's assessments.

## Results

**FIGURE 1:**
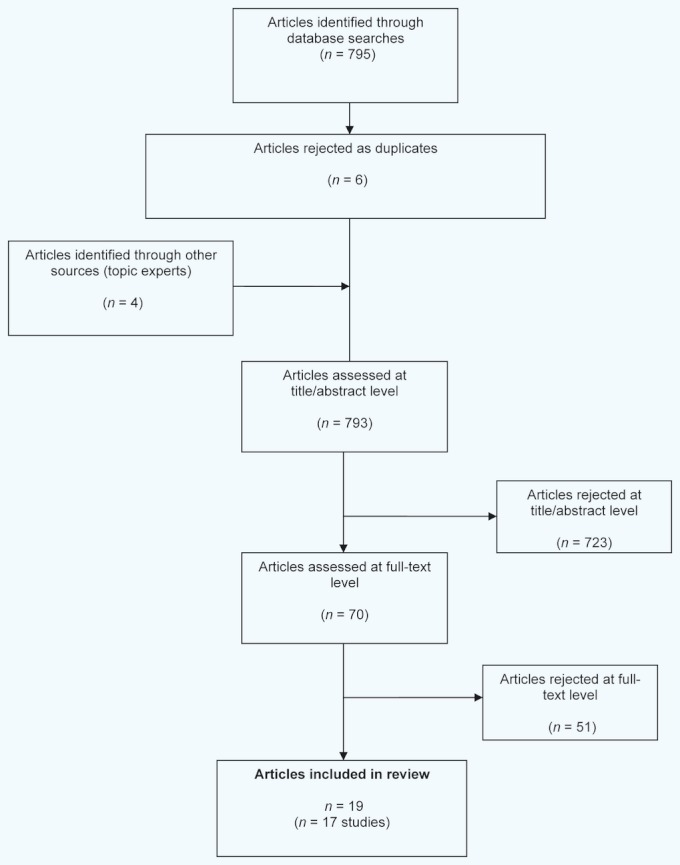
Flow chart of paper selection

The initial searching following de-duplication produced a total of 793 database citations, including four papers suggested by topic experts [13–16]. Of these, 723 were rejected at title/abstract level and 70 were considered at full text. Fifty-one studies were rejected at full-text level, with a total of 17 studies reported in 19 primary level papers assessed as appropriate for inclusion (see Fig. 1 and Table 1). One included study reported findings in two papers at 16-week and 10-month time points [13,17]. Another study reported findings in two papers at 1-year and 3-year follow-up [18,19].

**Table 1:** Characteristics of intervention studies based on the Diabetes Prevention Program (DPP) and the Diabetes Prevention Study (DPS)

Author, country, setting	Study design	Population	Intervention	Comparator(s)	Length of follow-up
Diabetes Prevention Program (DPP) [3,8] USA	Randomized controlled trial. Three groups	Adults with fasting plasma glucose 5.3–6.9 mmol/l; impaired glucose tolerance; BMI ≥ 24 kg/m^2^ (22 in South Asian population)	16 core sessions 30–60 min. Maintenance: phone or in person 1–2 times monthly	Standard 20- to 30-min session and written materials	3.2 years and 10 years
Ackermann *et al*., 2008 [22] USA Young Men's Christian Association (YMCA)	Pilot cluster—randomized trial (DEPLOY). Two groups	Semi-urban. American Diabetes Association risk score ≥ 10, BMI ≥ 24 kg/m^2^	16 group sessions (*n* = 8–12) of 60- to 90-min duration. Maintenance: twice weekly individual or group for 4 weeks. Monthly YMCA sessions open to family members	Brief counselling alone	12 months
Almeida *et al*., 2010 [24] USA Integrated health care	Non-randomized, longitudinal. Two groups (matched pairs)	Newly diagnosed pre-diabetes. Impaired glucose tolerance (100–125 mg/dl)	Two 4- to 6-monthly group sessions (*n* = 10–20). 90-min duration	Usual care	12 months
Amundsen *et al*., 2009 [17] Vanderwood *et al*., 2010 [13] USA Primary care	Evaluation. One group	BMI ≥ 25 kg/m^2^ One or more: impaired fasting glucose/impaired glucose tolerance; hypertension; hyperlipidaemia; history of gestational diabetes; birth to baby of > 4 kg	16 group sessions (*n* = 8–34) of 60-min duration. Maintenance: monthly sessions over 6–12 months	N/A	12 months
Boltri *et al*. 2008 [27] USA African American Baptist church	Pilot pre-test post-test. One group	Pre-diabetes (fasting glucose 100–125 mg/dl)	16 sessions over 4 months. Modified for church implementation. Culturally sensitive	N/A	12 months
Davis-Smith *et al*., 2007 [28] USA Rural African American church	Evaluation One group	Church congregation. American Diabetes Association risk score ≥ 10. Fasting blood glucose 100–125 mg/dl	Six sessions over 7 weeks	N/A	12 months
Faridi *et al*., 2010 [16] USA African American church	Evaluation. Two groups	African American church congregations. One or more: BMI > 25 kg/m^2^; parent/sibling with diabetes; history of gestational diabetes	Number of sessions not standardized. Flexible content and mode of delivery (either group or individual)	Intervention compared in two geographical settings	12 months
Katula *et al*., 2011 [15] USA	Randomized controlled trial (HELP-PD). Two groups	BMI between 25–40 kg/m^2^ Pre-diabetes identified on two occasions (HOMA-IR).	24 group sessions (weekly for 6 months). Maintenance: monthly sessions for 18 months	Two individual sessions with nutritionist over 3 months	24 months
Kramer *et al*., 2009 [30] USA	Non-randomized prospective. One-group	25–74 years BMI ≥ 25 kg/m^2^ Fasting glucose 100–125 mg/dl	12 group sessions of 1-h duration delivered over 12–15 weeks	N/A	12 months
Kulzer *et al*., 2009 [14] Germany	Randomized controlled trial (PREDIAS). Two groups	Age 20–70 years, BMI ≥ 26 kg/m^2^, impaired glucose tolerance or impaired fasting glucose. Diabetes Risk Score > 10	12 group sessions of ∼90 min. Eight core sessions over 8 weeks then every 2 weeks	Written information and materials	12 months
McTigue *et al*., 2009 [25] USA	Controlled cohort Study (WiLLoW). Two groups	BMI ≥ 25 kg/m^2^	12 group sessions. Maintenance: monthly sessions for 8 months	Individuals that did not enrol on the programme	12 months
McTigue *et al*., 2009 [29] USA Primary care	Cohort study. One group	Age 18–80 years. Access to Internet. BMI ≥ 25 kg/m^2^ History of cardiovascular disease risk factors (including pre-diabetes)	16 weekly 30- to 45-min online sessions. Maintenance: 8-monthly lessons	N/A	12 months
Seidal *et al*., 2008 [31] USA Medically underserved communities	Non-randomized prospective. One-group	BMI ≥ 25 kg/m^2^ ≥ 3 components of the metabolic syndrome	12 group sessions (*n* = 5–13) over 12–14 weeks. 90-min duration	N/A	6 months
Vadheim *et al*., 2010 [26] USA Hospital clinic and online	Non-randomized controlled trial. Two groups	Age > 21 years BMI ≥ 25 kg/m^2^ At least one diabetes/cardiovascular disease risk factor or history of gestational diabetes Birth to baby of > 4 kg	16 weekly tele-health sessions, 60 min in duration. Maintenance: 6 × monthly sessions	Hospital site: 2- to 4-weekly supervised physical activity sessions	16 weeks
Whittemore *et al*., 2009 [23] USA Nurse practitioner clinics	Pilot randomized controlled trial Two groups	Age ≥ 21 years BMI ≥ 25 kg/m^2^ If < 65 years: Family history of Type 2 diabetes History of gestational diabetes Birth to baby of > 4 kg Ethnic group at high risk	Six in-person 20-min sessions and five phone sessions over 6 months	One nurse practitioner and one nutrition session	6 months
Diabetes Prevention Study (DPS) [4,9] Finland	Randomized controlled trial. Two groups	Impaired glucose tolerance and BMI ≥ 25 kg/m^2^ *n* = 523 randomized *n* = 212 at 12 months	Seven sessions with nutritionist during first 12 months, followed by visits every 3 months	General information at start of trial	3 years
Absetz *et al*., 2007/2009 [18,19] Finland Primary care	Pre-test post-test (GOAL). One group.	Diabetes risk score ≥ 10	Six group counselling sessions (*n* = 12) over 8 months; 2 h duration. Follow-up measurements: years 1 and 3	N/A	1 and 3 years
Laatikainen *et al*., 2007 [21] Australia Primary care	Pre-test post-test Greater Green Triangle). Two groups	Diabetes risk score ≥ 12	Six group sessions over 8 months; 90-min duration. Five sessions in first 3 months; final session at 8 months	N/A	12 months
Saaristo *et al*., 2010 [20] Finland Primary care and occupational health	Pre-test post-test (FIN-D2D). One group	FINDRISC ≥ 15	Four to 9 group weekly or biweekly sessions and some individual sessions. Follow-up session 1 month after intervention	N/A	12 months

FINDRISC, Finnish Diabetes Risk Score; HELP-PD, Healthy Living Partnerships to Prevent Diabetes; HOMA-IR, homeostasis model assessment of insulin resistance; N/A, not available; PREDIAS, Prevention of diabetes self-management program; WiLLoW, Weight Loss through Living Well.

### Characteristics and quality

Generally, the quality of the included studies was moderate to good (see also Supporting Information, Appendix S2). No included study complied with all of the 27 quality criteria in the assessment tool [12], although this was mainly attributable to the range of study types included and the complexity of the intervention. For example, concealment of treatment type is unlikely to be feasible for lifestyle interventions.

We included all relevant studies, particularly as they provided results from a range of settings, with the caution that results from non-randomized controlled trials and observational studies are more likely to be impacted by bias. Whilst the individually randomized trials generally had very good internal validity, inevitably the more pragmatic and non-randomized studies were at greater risk of both selection and information biases and therefore had weaker internal validity (with potentially greater external validity), as discussed in the following section.

The studies varied in terms of sample size, length of follow-up and the presence of a comparator (see Table 1). All the included studies based their protocol on either the DPP [3] or the Finnish DPS [4]. One study based the interventions on both these protocols. No study was based on any of the other major diabetes prevention studies, such as the Da Qing [5] or the Indian Diabetes Prevention Programme [6].

Fourteen studies were carried out in the USA in a range of settings with protocols based on the DPP. Three studies were based on the DPS only; two carried out in Finland [18–20] and one in Australia [21]. A further German study [14] was based on both the DPP and the DPS. Four studies [14,15,22,23] were randomized controlled trials, although, of these, two [22,23] were pilot studies with small samples, and one had only a 6-month follow-up [23]. Four non-randomized studies compared findings between groups. One non-randomized study compared an intervention implemented in church settings with a control [16].

The Almeida study [24] compared matched pairs from a healthcare organization and one study compared outcomes from those that had not enrolled onto a programme with those that had enrolled [25]. Vadheim *et al*. [26] compared outcomes from two groups that received the same intervention in different settings. The remaining studies [13,17,19–21,27–29,31] used a pre-/post-test single-group design. Follow-up ranged from 16 weeks to 3 years, with 12 studies providing results from a follow-up of at least 12 months. Settings were mainly healthcare related, typically an outpatient clinic. Three studies [16,27,28] delivered the intervention through US churches, and one [22] used Young Men's Christian Association (YMCA) facilities compared with a healthcare setting. Two studies [26,29] used available technology to deliver the interventions, one via the Internet and one through videoconferencing (tele-health).

All the studies targeted ‘at-risk’ populations, with one or more risk factors for Type 2 diabetes, such as having a BMI of ≥ 25 kg/m^2^, having raised blood glucose levels and/or a raised diabetes risk score. The programmes included a dietary as well as a physical activity component to the intervention, as with the DPP and DPS protocols. As in the original trials, trained personnel such as nurses, dieticians and physical fitness experts were recruited to deliver the interventions in all but one study, where community members were trained to carry out a church-based intervention [16]. The included studies typically did not describe intervention content in detail, as they were based on the two protocols. There was generally an emphasis on goal-setting as well as self-monitoring dietary and physical activity achievements in order to achieve weight loss goals of between 5 and 7%.

Specific modifications to the original trial protocols were described. To allow feasibility in community settings in terms of resources, the most common modification was a decreased number of sessions. In addition, the tendency for individual-based visits and sessions in the DPP and DPS was altered to group sessions. Interventions were also modified to increase accessibility to the venue and to the intervention in terms of cultural sensitivity for the target audience. For example, three interventions utilized church premises from which the African-American congregation were invited to be screened or to receive the intervention. However, one of these studies reported that blood testing was not allowed on church premises [16].

Church-based strategies have been used in other screening programmes; as well as having the potential to increase uptake, original protocol materials are modified to increase cultural sensitivity for diverse groups. Another study used well-established networks developed through the Young Men's Christian Association to access a wider population, as well as to sustain interest through membership. Two studies harnessed tele-health technology (video-conferencing and the Internet) as methods of delivering lifestyle interventions to a wider population.

Studies varied in terms of sample characteristics, such as sex and ethnicity (see Table 2). Three studies targeted African-American populations. Ethnicity of the sample was not reported in any of the three DPS-based studies, nor in five of the DPP-based studies. Of the remaining DPP-based studies, all but one [29] included more than 25% non-white participants. The majority of participants in all but one study [14] were female.

**Table 2:** Findings from intervention studies based on the Diabetes Prevention Program (DPP) and Diabetes Prevention Survey (DPS)

Author, country, setting, follow-up	Sample	Changes in mean blood glucose at follow-up	Mean weight loss at follow-up	Change in mean waist circumference at follow-up	Lost to follow-up
Diabetes Prevention Program (DPP) [3,8] USA	Lifestyle intervention *n* = 589 (female 68%; non-white 53%) Control *n* = 582	Intervention: HbA_1c_ 41 mmol/mol (5.9%) to 40 mmol/mol (5.8%) Control: HbA_1c_ 41 mmol/mol (5.9%) to 42 mmol/mol (6.0%) at 12 months	7 kg over 12 months, then a gradual regain to 2 kg at 10 years	Not reported	Not seen in 18 months: 65 (11%) in lifestyle intervention arm 69 (11.9%) in control arm
Ackermann *et al*., 2008 [22] USA Young Men's Christian Association (YMCA) 12 months	*n* = 92 (46 intervention; 46 control) Non-white 29% Female 61%	Intervention: HbA_1c_ (−0.1%)Control: no change (*P* = 0.28)	Intervention: 6.0 kg Control: 1.8 kg (*P* = 0.008)	Not reported	15% intervention 17% control
Almeida *et al*., 2010 [24] USA Integrated health care 12 months	*n* = 1520 760 matched pairs Ethnicity not reported Female 53%	Not reported	Intervention: 1.4 kg (95% CI 1.6 kg to 1.1 kg) Control: 0.6 kg [95% CI 0.9 kg (2.0 lbs) to 0.4 kg (0.8 lbs)] (*P* < 0.001)	Not reported	60 from each arm (total 15.8%) at 12 months
Amundsen *et al*., 2009 [17] Vanderwood *et al*., 2010 [13] USA Primary care 12 months	*n* = 355 Ethnicity not reported Female 65%	Not reported Fasting blood glucose: −0.26 mmol/l (sd 0.39) at 10 months	6.7 kg (sd 4.0) no other details reported 9.5 kg (sd 19.3) at 10 months	Not reported Not reported	17.5% at 4 months
Boltri *et al*., 2008 [27] USA African American Baptist church	*n* = 26 All African American Female 58%	−0.22 mmol/l	0.45 kg at 12 months (2.52 kg at 6 months)	Not reported	None reported
Davis-Smith *et al*., 2007 [28] USA Rural African American church	*n* = 11 All African American Female 64%	Fasting serum glucose −0.5 mmol/l	4.0, 3.0 and 4.8 kg immediately after the intervention, and at 6- and 12-month follow-up, respectively. No other details reported	Not reported	*n* = 1 (9%)
Faridi *et al*., 2010 [16] USA African American church	13 congregations Intervention: *n* = 121 Comparator: *n* = 125 100% African American Female: 85% intervention, 72% control	Not reported	Intervention: +0.32 lbs (0.15 kg) (sd 25.92) Control: +0.82 lbs (sd 19.30) (0.37 kg)	Not reported	Intervention: *n* = 31% Comparator: *n* = 37.6% At 12 months No reasons
Katula *et al*., 2011 [15] USA 12 months	*n* = 301 Intervention: *n* = 151 Control: *n* = 150 25% African American Female 58%	−0.21 mmol/l (sd 0.02)	−2.57 kg (sd 0.42)	−5.05 cm (sd 0.38)	Attrition at 6 months: Intervention: 1% Control: 2% Attrition at 12 months: Intervention: 1% Control: 2%
Kramer *et al*., 2009 [30] USA 12 months	*n* = 51 phase 1 27% non-white Female 82% *n* = 42 phase 2 0% non-white Female 79%	−0.08 mmol/l or −1.4% (*P* = 0.52)	Phase 2: −4.5 kg (*P* < 0.001)	Phase 2: −4.3 cm (−1.7 inches) (−4.2%), (*P* < 0.001)	Phase 1: 18 (35%) did not attend post-assessment visit Phase 2: 2 (4.8%) did not attend post-assessment visit; 12 (28.6%) did not attend 12-month visit
Kulzer *et al*., 2009 [14] Germany 12 months	*n* = 182 Ethnicity not reported Female 43%	Fasting blood glucose −4.3 mg/dl (0.24 mmol/l) (sd 6.0) (*P* = 0.001) HbA_1c_ 0.0% Oral glucose tolerance test −7.3 mg/dl (0.4 mmol/l) (sd 30.8)	−3.8 kg (sd 5.2) (*P* < 0.001)	−4.1 cm (sd 6.0)	17 lost to follow-up (9%)
McTigue *et al*., 2009 [25] USA	*n* = 155 Enrollees *n* = 72 Non-enrollees *n* = 82 Ethnicity not reported Female 84%	Not reported	Intervention: 5.19 kg (95% CI −7.71 to −2.68) Control: +0.21 kg (95% CI −1.50 to 1.93) (*P* < 0.001)	Not reported	Follow-up data unavailable for 7% of sample [attrition *n* = 5 (6.9%) in enrollees; *n* = 16 (19.5%) in non-enrollees)
McTigue *et al*., 2009 [29] USA Primary care	*n* = 50 8% African American 4% Asian Female 76%	Not reported	4.79 kg (95% CI −7.36 to −2.22)	Not reported	Attrition: *n* = 5 (10%)
Seidal *et al*., 2008 [31] USA Medically underserved communities 6 months	*n* = 77 36% non-white Female 74%	Proportion with ≥ 5.5 mmol/l increased over time (baseline 42%; 3 months 51%; 6 months 61%; *P* = 0.06; adjusted *P* = 0.001)	46% lost ≥ 5% body weight; 26% lost ≥ 7% after 3 months. At 6 months this was sustained by 87 and 67%, respectively	Abdominal obesity decreased: Baseline 90%; 3 months 82%; 6 months 68% (*P* = 0.006)	10.4% attrition at 3 months; 35% at 6 months
Vadheim *et al*., 2010 [26] USA Hospital clinic and online 16 weeks	*n* = 13 on site *n* = 16 tele-health Ethnicity not reported Female 69%	Not reported	Intervention: 6.7 kg (sd 3.7) Control: 6.5 kg (sd 3.1) (*P* = 0.85)	Not reported	On site: 0% Tele-health: 12.5% at 16 weeks
Whittemore *et al*., 2009 [23] USA Nurse practitioner clinics 6 months	*n* = 58 Intervention: *n* = 31 Control: *n* = 27 Non-white 55% Female 93%	Intervention: trend in oral glucose tolerance test 0.01 mmol/l per month Control: 0.83 mmol/l per month	Intervention: 1.5% (*P* = 0.8) Control: 0.0% (*P* = 0.45)	Not reported	12% attrition at 6 months
Diabetes Prevention Survey (DPS) [4,9] Finland 12 months	*n* = 522 Female 66% Ethnicity not reported	Fasting plasma glucose. Intervention: −0.2 mmol/l (sd 0.31) (95% CI −6 to −2) Control: +0.05 mmol/l (sd 0.001) (95% CI 0–2) (*P* < 0.001)	Intervention: −4.2 kg(sd 5.1) Control: −0.8 kg (sd 3.7) (*P* < 0.001)	Intervention: −4.4 cm (sd 5.2) (95% CI 5.1–3.9) Control: −1.3 cm (sd 4.8) (95% CI 1.9–0.7) (*P* < 0.001)	59% attrition at 12 months
Absetz *et al*., 2007/2009 [18] Finland Primary care 12 months and 3 years	*n* = 352 |Ethnicity not reported Female 77%	Fasting plasma glucose: +0.1 mmol/l (sd 0.6) (*P* < 0.001) Oral glucose tolerance test: +0.1 mmol/l (sd 1.7) (NS) At 3 years: 0.01 mmol/l (sd 0.8) (NS) Oral glucose tolerance test: +0.1 (sd 1.9) (NS)	−0.8 kg (sd 4.5) (*P* = 0.002) At 3 years: −1.0 kg (sd 5.6) (*P* < 0.003)	−1.6 cm (sd 4.8) (*P* = 0.001) At 3 years: +0.1 cm (sd 6.4) (NS)	23% attrition at 3 years
Laatikainen *et al*., 2007 [21] Australia Primary care 12 months	*n* = 311 Ethnicity not reported Female 55%	Fasting plasma glucose: −0.14 mmol/l (95% CI −0.20 to −0.07) (−2.5%) Oral glucose tolerance test: −0.58 (95% CI −0.79 to −0.36) (−8.6%)	−2.36 (95% CI −3.19 to −1.85)	−4.17 cm (95% CI −4.87 to −3.48) (−4.0%) Some reduction in 75% of sample	74 (23.8%) non-completers
Saaristo *et al*., 2010 [20] Finland Primary care and occupational health 12 months	*n* = 2798 Ethnicity not reported Female 67%	Not reported	Males: −1.2 kg (sd 5.3) (*P* < 0.0001) Females: −1.1 kg (sd 5.8) (*P* < 0.0001)	Males: −1.3 cm (sd 4.9) (*P* < 0.0001) Females: −1.3 cm (sd 5.9) (*P* < 0.0001)	32% attrition

NS, not signifcant;

### Reported outcomes

The primary outcome of the DPP and DPS was cumulative diabetes incidence at follow-up. This outcome was not typically measured in translational studies that included a comparator, probably because there was not sufficient statistical power to do so. Therefore, it is difficult to make direct inferences about the effectiveness of the intervention in reducing diabetes. All studies reported changes in body weight, and some also reported blood glucose levels and waist circumference.

### Findings—weight change

All the included studies assessed changes in weight at baseline and follow-up (see Table 2). Included randomized controlled trials [14,15,22] reported greater weight loss (at least 4.0%) in the intervention arm than in the control groups (no greater than 2.0%). Whittemore *et al*. [23] reported ≥ 5% weight loss in 25% of the intervention group compared with 11% of the control group at 6 months.

Non-randomized studies also reported weight loss. The largest study [24] reported a loss of > 5% body weight that was 1.5 times more likely in the intervention arm. In the Weight Loss through Living Well (WiLLoW) study, 27% of enrollees achieved more than 7% weight loss compared with 6% of non-enrollees [25]. Motivation may, however, be higher in those that enrolled to the interventions. At 16 weeks, 48% of a tele-health intervention group and 50% of the comparator group achieved at least 7% weight loss, with the mean weight loss in both groups more than 6.4 kg [26]. In this study, the same intervention was being tested in two different settings.

In three studies that did not include comparators, the goal to lose at least 7% body weight was achieved by between 18 and 45% of participants at between 10 and 12 months [13,29,30]. Two church-based interventions achieved weight losses of 3.6 and 4.6%, respectively [27,28].

The three DPS-based studies achieved smaller weight losses at 12 months than did the DPP-based studies. The Australian study [21] achieved the greatest weight loss (2.7%). Absetz and colleagues [18,19] reported differences in weight loss for men and women (1.5 and 0.6%, respectively), whilst Saaristo *et al*. [20] reported the same reduction in both men and women (1.3%). The latter study reported sustained weight reduction at 3 years.

Only one non-randomized study reported no weight loss [16], with a mean gain in weight of 0.2% in the intervention arm and 0.4% in control subjects. However, there were reported significant differences in baseline characteristics of intervention and control groups.

### Findings—changes in waist circumference

Changes in waist circumference were reported in seven studies. In two randomized controlled trials, reductions of at least 4 cm were reported in the intervention arm compared with less than 0.6 cm in the controls after 12 months [14,15]. Single-group studies based on both the DPP and DPS also reported reductions of between 1.6 and 4.3 cm at 12 months [14,19,21,31], although in one study this was not sustained at 3 years [20]. Authors of one study [21] calculated that their reported 4.0% reduction in waist circumference equated with a 40% reduction in diabetes risk.

### Findings—changes in blood glucose and HbA_1c_ levels

Reported changes in blood glucose following DPP- or DPS-based interventions were minimal. However, caution needs to be taken in interpreting these figures as measuring average changes in blood glucose can mask some instances of significant reduction.

Two randomized controlled trials [14,15] each reported mean decreases in blood glucose of 0.24 mmol/l in the intervention groups at 12 months' follow-up compared with 0.02 mmol/l and 0.09 mmol/l, respectively, in the control groups (*P* < 0.001).

The Kulzer study [19] found no mean change in HbA_1c_ at baseline and 12 months in the intervention group, and a rise of 22 mmol/mol (approximately 2.0%) in the control group (*P* = 0.165). Ackermann and colleagues [22] reported a reduction in mean HbA_1c_ of 0.1% compared with no change in the control subjects (*P* = 0.28) at 12 months.

No other included studies that measured blood glucose levels provided a comparative measure. The largest mean reduction in fasting blood glucose at 12 months was 0.5 mmol/l. This was achieved following a US church-based intervention [28]. A reduction of 0.26 mmol/l was reported in primary care settings [17].

Similarly, interventions based on the DPS reported minimal mean change in fasting plasma glucose at 12 months (+0.1 mmol/l and −0.14 mmol/l) [18,21], and at 3 years 0.0 mmol/l [19]. These limited results could be attributable to a regression to the mean over time.

Seidal and colleagues [31] reported an increase in the number of participants from low socio-economic groups that had fasting blood glucose levels equal to or above 5.5 mmol/l at 3- and 6-month follow-up.

### Reported associations between outcomes

#### Weight change and programme delivery

One church-based study [16] attributed their negative weight change results to a lack of fidelity to the DPP protocol; community members were trained to deliver the intervention rather than utilizing qualified health professionals and experts. The trained advisors were encouraged to be creative regarding the content and mode of delivery of the intervention, tailoring to each individual participant's preferences.

#### Weight change and programme attendance

The Saaristo *et al*. study [20] reported that those who lost more than 5% of their body weight made on average the most intervention visits (3.5), whilst those that maintained their weight made an average of 2.9 visits, and those that gained weight made 2.5 visits. However, this result could be confounded by the higher baseline weight of those that made the most visits (BMI 32.6 ± 5.6 kg/m^2^ compared with 31.3 ± 5.1 kg/m^2^ for those making two visits, 30.7 ± 4.8 kg/m^2^ for those making one visit and 30.9 ± 5.0 kg/m^2^ for those making no visits). In addition, Ackermann and colleagues reported a 6.0% reduction in weight despite 57% overall attendance [22].

#### Weight change and diabetes incidence

Saaristo *et al*. [20] analysed weight change and diabetes incidence in their DPS-based study, reporting a stepwise association. Incidence in those that lost more than 5% of body weight over 12 months was 2% compared with those that gained weight (almost 8%), and those that maintained a stable weight (7%). Incidence was also more likely at 12-month follow-up in those that already had impaired fasting glucose or impaired glucose tolerance at baseline, with a 6- to 9-fold increase in likelihood of developing diabetes than those with normal glucose levels at baseline.

#### Waist circumference and diabetes incidence

One Australian study [21] used results from the DPS sample as a reference to equate their reported 4.0% reduction in waist circumference with a 40% reduction in diabetes risk.

### Loss to follow-up

The majority of studies lost between 9 and 15% of the original sample during follow-up. However, there were cases of very high and low attrition rates. All the DPS-based studies reported at least 23% attrition over 1 or 3 years and one church-based study lost a third of the small sample over 12 months [16]. By contrast, one sizeable and diversely populated randomized controlled trial reported loss to follow-up of 2% or less in both arms [15]. This would suggest that findings from this study could be considered as relatively robust.

## Discussion

This review aimed to assess the impact of adapting diabetes prevention protocols to ‘real-world’ settings on key outcomes. We included 19 studies (reported in 17 papers) based on either or both the DPP and the DPS protocols in terms of aims and content. Interventions were adapted to a range of settings and modified for feasibility.

The main modifications were number of sessions and mode of delivery (i.e. group-based rather than one-to-one) to reduce the resources required and also to increase accessibility to diverse populations. Some of the sample sizes were very small and some follow-ups were short; only one study provided results beyond 12 months' follow-up. Seven studies included a comparator; four were randomized controlled trials, of which two were pilot studies.

Populations in the DPP-based studies were more diverse, including up to 100% of non-white participants. However, there was no particular distinction made in outcomes between ethnic groups. Most of the interventions attracted predominantly women, highlighting the need to address ways of increasing the accessibility and acceptability of lifestyle intervention for men.

Reporting of weight loss outcomes differed between studies and included mean weight reduction, percentage weight reduction or the percentage achieving a specified weight loss. Much of the detail regarding delivery of interventions was not reported. This degree of heterogeneity was deemed not appropriate for a meta-analysis.

Whilst the findings varied widely in terms of effect size, there was a strong trend toward weight loss following all but one of the interventions. In one study, over 45% of participants who had completed the intervention achieved the goal of at least 7% weight reduction [13]. This figure may, however, be inflated compared with those derived from an intention-to-treat analysis such as that carried out in the DPP. Studies that included a comparator reported greater effects in the intervention arm than in the control subjects. One study reported no weight reduction [16] although the intervention arm gained less weight than the control. This could be attributable to significant differences in baseline characteristics as well as lack of protocol fidelity.

Changes in waist circumference were not reported in all studies, although, in the seven studies that did, this outcome was favourable and was associated (through imputation) in one study with a reduction in diabetes risk [21]. A minority of studies measured mean fasting blood glucose or HbA_1c_ changes, with reported reductions mirrored by the DPP where 1% change was detected at 12 months.

Reduction in diabetes incidence was not measured in any controlled study. This may reflect the difficulty assessing incidence within the short duration of the included studies. One study, however, provided evidence of a stepwise incidence associated with weight gain [20]. This suggests that Type 2 diabetes can be prevented in ‘real-world’ settings, providing there is sustained weight management. The main challenge is how best to deliver and achieve engagement with interventions and how to sustain lifestyle change. Attrition rates varied across the studies from very low to approximately one third of participants. This has to be taken into account in terms of assessing the robustness of findings, as well as reasons for participant dropout and whether these can be addressed in future programmes.

Large randomized controlled trials have shown that the onset of Type 2 diabetes can be prevented or delayed to a large extent, particularly in those that achieve lifestyle targets [8]. One review of behavioural strategies [32] highlighted individualized delivery as a factor in the success of large randomized controlled trials. One-to-one intervention delivery, however, is unlikely to be feasible in clinical practice because of resource and financial restraints. This review demonstrates that group-based interventions can yield significant weight loss (with the expectation of reductions in the risk of Type 2 diabetes), provided that changes are sustained over a number of years. Even in the intensive DPP [3] and DPS trials [4], there was partial regain of weight in the intensive arm. It is clear from reviews of behavioural strategies that effectiveness reflects intensity of the intervention, as well as proven behavioural strategies, and that interventions comprising modified versions of most of the core modules of the original DPP are the most effective [7,32].

The feasibility of implementing nationwide diabetes prevention programmes is dependent on identifying the most economic modes of delivery. Findings from this review suggest that significant weight loss may be achievable with larger groups than are currently adopted in clinical practice, with some DPP translation studies using classes of 15 [24] and 17 participants [17]. Equally important is the skill of the educators [34]. There was a variety of professional backgrounds amongst the educators in the studies in this review, with associated variation in costs. Further research is needed to identify the most cost-effective mode of delivery. From the findings of the included papers, one option may be a highly qualified diet and physical activity professional supported by a less-qualified individual.

Other reviews have assessed the translation of diabetes prevention studies into ‘real-world’ settings. One review identified 12 studies that were all carried out in clinical settings. Not all the included studies stated that they were based on a particular protocol such as the DPP or the DPS. Results from four studies that were meta-analysed showed a positive effect on weight and waist circumference at 1 year [35]. Our review supports the findings that significant effects from translational lifestyle interventions on clinical parameters such as blood glucose and diabetes risk may be difficult to demonstrate, and that decreases in weight following adapted interventions are a more promising finding [36]. Another review assessed studies based on the DPP and translated into community settings such as churches [36]. The review included seven papers, although four of these did not exclude individuals with known Type 2 diabetes at baseline. The authors conclude that significant weight loss was achieved in three studies at 12 months following intensive interventions [36].

This review has assessed only those studies that applied a specified, known protocol that has previously been associated with a reduction in the incidence of Type 2 diabetes as well as weight loss. Given the relatively short follow-up and smaller sample size, translational studies were more likely to have sufficient statistical power to measure change in weight than in diabetes incidence. However, weight loss is associated with a reduction in diabetes incidence. In the DPP, for example, weight loss was reported to be the dominant factor in diabetes incidence reduction in a high-risk population, to the extent that 5 kg lost was estimated to result in a 55% reduction in incidence over 3 years' follow-up [37].

Some of the interventions may be regarded as country or health system specific. However, the general principles of lifestyle change to reduce weight and diabetes incidence are generalizable to any high-risk population. Some issues to consider in translating diabetes prevention trials into community settings include the extent of available resources, differences between healthcare delivery organizations and cultural variations between settings. For example, whilst the Young Men's Christian Association network might provide a useful gateway from which to access at-risk populations in the USA, this might not be the case elsewhere. In addition, church-based interventions were specific to the African-American population. To increase applicability to a specific setting, the intervention would require to be tailored to meet the needs of local faith groups. Future research should assess whether translating prevention protocols is feasible in terms of acceptability and cost.

## Conclusions

Translational studies based on the DPP and the DPS, but with modifications to increase feasibility, reported mean and percentage weight loss (as well as reductions in waist circumference) in a range of settings. Weight loss is associated with diabetes prevention and so can be regarded as a marker for potential prevention over the longer term, although current evidence for sustainability beyond 3 years is limited. There is therefore potential, given that the lower costs of group-based interventions lessens financial barriers to implementation, for interventions to have an impact on future progression to diabetes in at-risk individuals in ‘real-world’ settings. More long-term research is required to assess the sustainability and long-term outcomes of translational interventions.

## Funding sources

The National Institute for Health and Clinical Excellence.

## Supporting Information

**Appendix S1.** Search strategies.

**Appendix S2.** Quality assessment.
