# Supplementary material for: Can diabetes prevention programmes be translated effectively into real-world settings and still deliver improved outcomes? A synthesis of evidence
Source: Diabet Med. 2012 Dec 13;30(1):3–15. doi: 10.1111/dme.12018 (PMC3555428; doi:10.1111/dme.12018)
Supplement: Supplementary file 1 [file dme0030-0003-SD1.doc]

**Appendix 1: Search strategies**

**Overarching Search**

Sample search Ovid MEDLINE(R) In-Process & Other Non-Indexed Citations and Ovid MEDLINE(R) 1950 to Present

1. *prediabetic state/

2. (pre-diabetes or pre diabetes or raised glucose intolerance or impaired glucose level$ or impaired glucose tolerance or IGT or impaired fasting glucose or IFG or FPG or fasting plasma glucose or impaired glucose regulation or impaired glucose metabolism or raised glycated haemoglobin or raised glycated hemoglobin or high glycated Hb or hyperglycaemia or hyperglycemia).ti.

3. (prevention adj3 (type II diabetes or type 2 diabetes or T2D)).ti.

4. 1 or 2 or 3

5. *body mass index/

6. *obesity/

7. (south asia$ or black africa$ or black caribbean$ or pakistan$ or bangladesh$ or india$ or ethnic minorit$ or chinese or obes$ or waist circumference or "bmi > 3?" or BMI).ti.

8. 5 or 6 or 7

9. *Hemoglobin A, Glycosylated/ or *Mass screening/ or *Risk assessment/

10. (((risk assessment or monitoring or screening) adj2 diabetes) or HBA1C).ti.

11. 9 or 10

12. *Exercise/ or *Exercise therapy/ or *Diet therapy/

13. (lifestyle intervention$ or slimming club$ or diet or low glycaemic index or low glycemic index or reduced fat or low carbohydrate or low calorie or physical activit$ or exercise or cardiorespiratory training).ti.

14. (Motivational support adj5 diet).ti,ab.

15. 12 or 13 or 14

16. 8 or 11 or 15

17. 4 and 16

**Translational studies**

1 ((prevent$ or reduc$ or protect$ or limit$ or control$ or delay$) adj5 (diabetes or prediabetes or pre diabetes or raised glucose intolerance or impaired glucose level$ or impaired glucose tolerance or IGT or impaired fasting glucose or IFG or FPG or fasting plasma glucose or impaired glucose regulation or impaired glucose metabolism)).ti,ab.

2 *prediabetic state/

3 (prevent$ or reduc$ or protect$ or limit$ or control$ or delay$).ti,ab.

4 2 and 3

5 *Diabetes Mellitus, Type 2/pc

6 1 or 4 or 5

7 (DPP or diabetes prevention program or NIH-DPP or Finnish Diabetes Prevention Study or China Da Qing Diabetes Prevention Study or STOP-NIDDM).ti,ab.

8 6 or 7

9 (translat* or real world or adap*).ti,ab.

10 *Translational Research/

11 9 or 10

12 8 and 11

13 limit 12 to (english language and humans and yr="1990 - 2011")
