# Supplementary material for: Can diabetes prevention programmes be translated effectively into real-world settings and still deliver improved outcomes? A synthesis of evidence
Source: Diabet Med. 2012 Dec 13;30(1):3–15. doi: 10.1111/dme.12018 (PMC3555428; doi:10.1111/dme.12018)
Supplement: Supplementary file 2 [file dme0030-0003-SD2.doc]

**Appendix 2: Quality assessment**

1. Is the source population or source area well described?
2. Is the eligible population or area representative of the source population or area?
3. Do the selected participants or areas represent the eligible population or area?
4. How was selection bias minimised?
5. Were interventions (and comparisons) well described and appropriate?
6. Was the allocation concealed?
7. Were participants and/or investigators blind to exposure and comparison?
8. Was the exposure to intervention and comparison adequate?
9. Was contamination acceptably low?
10. Were the other interventions similar in both groups?
11. Were all participants accounted for at study conclusion?
12. Did the setting reflect usual UK practice?
13 Did the intervention or control comparison reflect usual practice?
14. Were outcomes measures reliable?
15. Were all outcome measurements complete?
16. Were all the important outcomes assessed?
17. Were all outcomes relevant?
18. Were there similar follow up times in exposure and comparison groups?
19. Was follow-up time meaningful?
20. Were exposure and comparison groups similar at baseline?
21. Was intention to treat (ITT) analysis conducted?
22. Was the study sufficiently powered to detect an intervention effect (if one exists)?
23. Were the estimates of effect size given or calculable?
24. Were the analytical methods appropriate?
25. Was the precision of intervention effects given or calculable? Were they meaningful?
26. Are the study results internally valid (i.e. unbiased)?
27. Are the findings generalisable to the source population (i.e. externally valid)?

**Quality assessment ratings by study (studies in bold include a comparator).**

| Study | 1 | 2 | 3 | 4 | 5 | 6 | 7 | 8 | 9 | 10 | 11 | 12 | 13 | 14 | 15 | 16 | 17 | 18 | 19 | 20 | 21 | 22 | 23 | 24 | 25 | 26 | 27 | Quality |
| --- | --- | --- | --- | --- | --- | --- | --- | --- | --- | --- | --- | --- | --- | --- | --- | --- | --- | --- | --- | --- | --- | --- | --- | --- | --- | --- | --- | --- |
| Absetz 2007; 2009 | ++ | ++ | + | NA | ++ | NA | NA | + | NA | + | + | NA | NA | ++ | + | ++ | ++ | NA | ++ | NA | NR | NR | + | + | + | NR | + | + |
| **Ackermann 2008** | **++** | **++** | **+** | **-** | **++** | **NA** | **NA** | **++** | **NR** | **+** | **+** | **NA** | **+** | **++** | **++** | **++** | **+** | **++** | **+** | **+** | **NR** | **NR** | **+** | **+** | **+** | **-** | **+** | **+** |
| **Almeida 2010** | **++** | **++** | **+** | **+** | **++** | **NA** | **NR** | **++** | **NR** | **++** | **++** | **NA** | **++** | **++** | **++** | **++** | **++** | **+** | **+** | **++** | **NR** | **+** | **+** | **+** | **+** | **+** | **+** | **++** |
| Amundsen 2009 / Vanderwood 2010 | ++ | ++ | + | NA | ++ | NA | NA | + | NA | NA | + | NA | + | ++ | ++ | ++ | ++ | NA | + | NA | NR | NR | + | + | - | + | + | + |
| Boltri 2008 | ++ | + | + | NA | + | NA | NA | + | NR | NA | ++ | NA | + | ++ | + | ++ | ++ | NA | + | NA | + | NR | - | + | - | - | + | + |
| Davis-Smith 2009 | ++ | + | + | - | + | NA | NA | NA | NA | NA | ++ | NA | + | + | ++ | + | ++ | NA | + | NA | NR | NR | - | + | - | - | + | + |
| **Faridi 2010** | **+** | **+** | **+** | **-** | **-** | **NA** | **NA** | **NR** | **NR** | **+** | **-** | **NA** | **+** | **+** | **+** | **+** | **++** | **+** | **+** | **-** | **NR** | **-** | **+** | **+** | **+** | **-** | **+** | **-** |
| **Katula 2011** | **+** | **++** | **+** | **NR** | **++** | **NA** | **NA** | **++** | **NR** | **++** | **++** | **NA** | **+** | **++** | **++** | **++** | **++** | **++** | **+** | **++** | **+** | **+** | **+** | **+** | **+** | **+** | **+** | **++** |
| Kramer 2009 | ++ | + | + | NA | ++ | NA | NA | NA | NR | NA | + | NA | + | **+** | **++** | **++** | **++** | NA | + | NA | **+** | **+** | **+** | **+** | **+** | - | + | + |
| **Kulzer 2009** | **+** | **+** | **+** | **++** | **++** | **NA** | **NA** | **++** | **NR** | **+** | **NR** | **NA** | **+** | **++** | **++** | **++** | **++** | **++** | **+** | **++** | **+** | **+** | **+** | **+** | **-** | **+** | **+** | **++** |
| Laatikainen 2007 | + | + | + | NA | + | NA | NA | + | NR | NA | ++ | NA | + | **++** | **++** | **++** | **++** | NA | + | NA | NR | **+** | **+** | **+** | **+** | - | + | + |
| **McTigue 2009a** | **+** | **+** | **+** | **-** | **+** | **NA** | **NA** | **+** | **NR** | **+** | **++** | **NA** | **+** | **-** | **+** | **++** | **++** | **-** | **+** | **+** | **NR** | **NR** | **-** | **+** | **-** | **-** | **+** | **+** |
| McTigue 2009b | + | + | + | NA | ++ | NA | NA | + | NR | NA | + | NA | + | + | + | ++ | ++ | NA | + | NR | NR | + | **+** | **+** | **+** | - | + | + |
| Saaristo 2007; 2010 | ++ | + | + | NA | - | NA | NA | + | NR | NA | + | NA | + | ++ | + | ++ | ++ | NA | + | NA | NR | NR | **+** | **+** | **+** | - | + | + |
| Seidal 2008 | ++ | + | + | NA | ++ | NA | NA | ++ | NR | NA | ++ | NA | + | + | ++ | ++ | + | NA | + | NA | - | NR | + | + | - | - | + | + |
| **Vadhelm 2010** | **++** | **+** | **+** | **NA** | **++** | **NA** | **NA** | **-** | **NR** | **NA** | **++** | **NA** | **+** | **-** | **++** | **+** | **++** | **NA** | **-** | **+** | **+** | **NR** | **-** | **+** | **-** | **-** | **+** | **+** |
| **Whittemore 2009** | **+** | **+** | **+** | **+** | **++** | **NA** | **NA** | **+** | **NR** | **+** | **++** | **NA** | **+** | **+** | **+** | **+** | **++** | **++** | **+** | **+** | **+** | **NR** | **+** | **+** | **-** | **-** | **+** | **++** |

NR = Not Reported, NA = Not Applicable
